# Factors associated with nursing students’ satisfaction with simulation-based assessments: interaction between psychological safety and gender in a cross-sectional study

**DOI:** 10.1186/s12909-025-07648-z

**Published:** 2025-07-14

**Authors:** Minjung Kyung, Hieun Kim, Jung-Hee Kim

**Affiliations:** College of Nursing, The Catholic University of Korea, 222 Banpo-daero Seocho-gu, Seoul, 06591 Korea

**Keywords:** Gender differences, Psychological safety, Simulation-based learning, Simulation-based assessments, Nursing student

## Abstract

**Background:**

Simulation-based learning (SBL) provides realistic clinical scenarios within a safe and controlled environment, allowing students to develop clinical skills without posing risks to patient safety. However, heightened anxiety surrounding performance assessments may hinder nursing students’ learning outcomes, with potential gender-based differences. Identifying factors associated with student satisfaction in SBL is crucial to optimize learning experience and improve educational strategies.

**Objectives:**

This study aimed to identify factors associated with satisfaction in SBL, and to examine the interaction between psychological safety and gender on satisfaction among nursing students.

**Methods:**

This cross-sectional study collected data from nursing students after simulation-based assessments. Based on the NLN/Jeffries Simulation Framework, multiple linear regression was employed to analyze the relationships between satisfaction in SBL and variables such as demographics, psychological safety, and simulation design features.

**Results:**

Among 243 nursing students in this study, 85.2% were female. In simulation-based assessments, female students were more sensitive to interpersonal risks in the simulation environment, whereas male students were more affected by high-fidelity design features. Higher psychological safety and better simulation design features were significantly associated with satisfaction in SBL. No significant interaction effect between psychological safety and gender was found.

**Conclusions:**

This study expands on prior research by providing empirical evidence of gender-specific responses to psychological safety and simulation design in SBL. The findings suggest the need for gender-sensitive approaches in SBL. This study might help guide educators and researchers develop effective simulation-based curriculums and provide foundational evidence for a professional development plan.

**Clinical trial number:**

Not applicable

## Background

Simulation-based learning (SBL) has attracted significant attention in nursing education as it provides an immersive and hands-on learning environment that closely mirrors clinical settings [1]. By offering a structured and controlled space for skill acquisition, critical thinking, and decision-making, SBL enables students to practice and refine their competencies without posing any harm to patients [2]. Students can enhance their knowledge, confidence, and professional readiness via repeated practice, constructive feedback, and reflective learning experiences [1, 2]. As patient acuity increases and access to clinical practice becomes limited, particularly in the context of the COVID-19 pandemic, SBL has emerged as an ideal for nursing education [3].

Simulation-based assessment serves as an effective approach to evaluating students’ cognitive, psychomotor, and affective competencies [4, 5]. Unlike traditional assessments that primarily measure isolated knowledge recall, simulation-based assessment facilitates a multidimensional evaluation of essential skills such as critical thinking, coping skills, knowledge of ethical considerations, technical skills, attitudes, and behaviors, by providing realistic clinical scenarios [6]. These assessments enable a more comprehensive and authentic appraisal of students’ preparedness for real-world nursing practice [6]. Simulation-based assessments can be integrated into both formative assessments and summative assessments [4]. Formative assessment are low-stake evaluations designed to provide ongoing feedback, allowing students and instructors to make real-time assessments [4]. In contrast, summative assessments are high-stake evaluations conducted at the end of a course to measure competency attainment and determine academic progression or certification eligibility [4].

Simulation-based assessments can also introduce significant psychological stressors [7]. The evaluative nature of these assessments—where students must demonstrate clinical competence under observation—can heighten anxiety, fear of failure, and self-doubt [7]. Research indicates that students experience greater stress and lower satisfaction when simulations are used for summative assessments rather than for formative learning [8]. A study of nursing students found that those who underwent summative simulation assessments reported significantly lower satisfaction compared to those who participated in formative assessments, highlighting the impact of evaluative pressure on student experiences [8].

One of the most critical but often overlooked factors in simulation-based assessments is psychological safety—the belief that individuals can engage in learning and assessment without fear of negative repercussions for their words, comments, or actions [9]. Psychological safety is particularly crucial in simulation-based assessments, where errors are visible and evaluations are explicit [9]. According to Bandura’s Self-Efficacy Theory, individuals’ confidence in their ability to succeed is shaped by past experiences, social reinforcement, and exposure to performance evaluations [10]. Students with low self-efficacy, often influenced by prior failures or negative feedback, may experience high anxiety and hesitation, leading to defensive learning behaviors such as reluctance to engage, hesitancy in decision-making, and avoidance of feedback [10]. In simulation-based assessments, this can manifest as student withdrawing from participation or struggling to perform under evaluative pressure.

However, psychological safety may not be experienced uniformly across genders. Research on psychological safety in workplace and educational settings suggests that gender differences exist in perceptions of risk, fear of evaluation, and responses to performance feedback [11, 12]. Women, in particular, may report greater anxiety and self-doubt in evaluative settings, which could influence their experiences in simulation-based assessments [11, 12]. In contrast, men may exhibit lower fear of failure and be more likely to engage in more risk-taking behaviors [11, 12]. Since psychological safety directly influences engagement, stress responses, and overall performance, these gender-based differences may lead to distinct experiences and outcomes in the same assessment environment.

Despite the potential significance of these differences, most prior research on SBL and psychological safety has focused on general student experiences, with limited attention to gender-specific mechanisms [13, 14]. To address this gap, this study aimed to: (1) investigate gender-based differences in psychological safety, simulation design features, and SBL satisfaction; (2) identify factors associated with satisfaction based on the NLN/Jeffries Simulation Framework [15] and (3) examine the interaction effect between psychological safety and gender on satisfaction and gender subgroup analysis following simulation-based assessments.

## Methods

### Study design and sample

This study was a cross-sectional analysis of a sample of 243 undergraduate nursing students from a single university in Seoul, South Korea. Fourth-year students who had completed an integrated clinical simulation course in the first semester were eligible for participation to minimize variability in baseline knowledge and skills. A power analysis using the G*Power 3.1.9.7 program indicated that 226 students were needed to achieve a medium effect size of 0.10, with a significance level of 0.05 and a power of 0.95, considering seven predictors. A total of 250 nursing students participated in the study, with an overall response rate of 100%. Of these, seven students were excluded from the analysis due to missing responses in the sections on psychological safety, simulation design features, and satisfaction following simulation-based assessments.

### Data collection

Data collection was conducted after the completion of integrated clinical simulation course in the first semester, spanning from 2022 to 2024. During this period, three cohorts of students completed the same standardized curriculum to ensure consistency across study years. In 2022 and 2023, data were collected using paper-based answer sheets, which students completed in pencil immediately following the course. In 2024, data collection was conducted online at the end of the course to align with institutional digitalization efforts. The professor in charge of the course and a teaching assistant administered the surveys, collected responses, and managed the data entry process for course evaluations. Student course evaluations were conducted immediately upon course completion to capture their real-time perceptions and experiences, minimizing recall bias. To reduce potential confounding effects, all students were randomly assigned to simulation groups and followed the same simulation scenarios, instructional methods, and assessment criteria across cohorts.

### Integrated clinical simulation course

We developed the course based on the stepwise model for simulation-based curriculum development [16]. The team developed simulation scenarios collaboratively and conducted a pilot test with a small group of recent nursing graduates to refine the course structure and content prior to its implementation. The integrated clinical simulation course was used to evaluate nursing competencies during a regular class period with one credit hour. As part of the regular curriculum at our institution, exit testing of senior nursing students is performed at the completion of all required course work in the undergraduate nursing program of the Catholic University of Korea. Summative simulation-based assessments were conducted. In compliance with the HSSOBP: Evaluation of Learning and Performance [4], evaluation training was provided to a total of 11 facilitators. The evaluation process began with a pre-briefing session, designed to familiarize students with the environment and equipment, and to alleviate any concerns or anxiety. Assessments were conducted by facilitators and video recordings were performed. The assessment tools were developed by the course team and their content validity was reviewed by subject matter experts. We also conducted a pilot test with 5–7 recent graduates from the school of nursing. Based on expert feedback and pilot results, the assessments were revised to improve clarity and alignment with course objectives.

After the assessments, a debriefing session was held. Performance of students in all simulated scenarios was assessed using checklists developed by instructors and validated by clinical experts. The instructors assessed the nursing students’ performances by administering two simulation scenarios per day. Students completed 30 h of simulation-based practice, covering eight scenarios over one week, focusing on critical thinking, communication, professionalism, teamwork, and coordination in various clinical situations. A total of eight scenarios based on various clinical environments were developed by the faculty in accordance with the learning goals at the time of curriculum development in 2021. Depending on the scenario, one to three students participated as a group, performing roles as either a primary or a secondary nurse. The simulations utilized a high-fidelity human patient simulator (i.e., a computerized manikin capable of replicating physiological response such as heart rate, breath sounds, and pupil reactions), a standardized patient (i.e., a trained individual portraying a patient case), or a hybrid method combining both approaches.

### Measures

The survey questionnaire for course evaluation comprised 54 items, with variables selected based on the NLN/Jeffries Simulation Framework [15]. This framework includes five components: participant, facilitator, educational practices, simulation design characteristics, and outcomes [15]. The facilitator component was not assessed in this study because as part of the regular curriculum, all students experienced the same learning conditions. We assessed the students’ general characteristics as a participant variable, psychological safety as an educational practice variable, and educational satisfaction as an outcome variable.

To ensure the applicability of the previously validated instrument to the new target population, content validity was reviewed by five experts. The external experts included two nursing professors, three clinical practice instructors with more than 3 years of experience. The content validity index for each scale item was calculated using a four-point Likert scale where 1 = not relevant and 4 = highly relevant. A pilot test was conducted to evaluate item comprehension among 10 senior nursing students in South Korea. The results of the preliminary investigation indicated that no expressions or questions posed difficulties for the respondents. Consequently, the final tools were completed without any modifications to the questions. For Exploratory Factor Analysis, principal component analysis with the varimax rotation was performed to identify the underlying structure of each scale.

#### Demographic characteristics

Demographic characteristics included gender, age, self-rated grade, and survey year. Students were asked to rate their academic performance by categorizing their grade as high, middle, or low.

#### Psychological safety

Psychological safety was assessed using the Psychological Safety Questionnaire in High Fidelity Simulations [17]. This 14-item questionnaire includes four subscales: (1) dealing with uncertainty (4 items), (2) being exposed (4 items), (3) being unsupported (3 items), and (4) interpersonal risk (3 items). Responses were measured on a 5-point Likert scale (1 = strongly disagree to 5 = strongly agree), with higher scales indicating greater psychological safety. To calculate the overall psychological safety score, item responses were summed to yield a total score ranging from 14 to 70. Reverse coding was applied to items where necessary to ensure that higher scores consistently represented greater psychological safety.

The content validity index (CVI) was calculated for each item (I-CVI), and all of the I-CVI values were 1.00. The scale-level CVI (S-CVI) was also 1.00, indicating excellent content validity. A pilot test was conducted with 10 nursing students, who rated the clarity of each item on a 4-point scale, with average scores ranging from 3.7 to 4.0, indicating that all items were generally well understood and easy to answer. The original measure, developed by Park and Kim, has demonstrated excellent reliability and good validity, with a Cronbach’s alpha of 0.91 [17]. Exploratory factor analysis (EFA) conducted by the original developers confirmed a four-factor structure, with factor loadings ranging from 0.635 to 0.850 and a total explained variance of 72% [17]. Our EFA results further confirmed the four-factor structure, with factor loadings ranging from 0.595 to 0.820 and a total explained variance of 69%. In the present study, the measure also showed strong internal consistency, with a Cronbach’s alpha of 0.81.

#### Simulation design feature

Simulation design was assessed using the Korean version of the Simulation Design Scale, developed by NLN [18, 19]. This scale consists of 21 items across five subscales: objective and information (6 items), support (4 items), problem solving (5 items), feedback/guided reflection (4 items), and fidelity (2 items). Items were rated on a 5-point Likert Scale (1 = strongly disagree to 5 = strongly agree). Simulation design score was calculated by summing the item responses, with total scores ranging from 21 to 105. Higher score represented increased recognition of simulation design features.

The content validity index was assessed, yielding perfect scores for all items, with both I-CVI and S-CVI recorded as 1.00, reflecting excellent agreement among experts. Results from the pilot test showed that item clarity was rated between 3.4 and 4.0 on a 4-point scale, indicating a high level of comprehension and ease of interpretation across all items.

The original measure has demonstrated good reliability and validity, with a Cronbach’s alpha of 0.92 [18]. The translated Korean version also showed strong internal consistency in previous studies (Cronbach’s alpha = 0.90) [19]. In this study, Cronbach’s alpha for this measure was 0.93. EFA revealed a five-factor structure, with factor loadings ranging from 0.534 to 0.921, collectively accounting for 68% of the total variance.

#### Satisfaction with simulation

Satisfaction following simulation-based assessments was measured using the 16-item Education Satisfaction Scale in Simulation for nursing students, developed by Kim and Heo [20]. The scale comprises three subscales: learning content (6 items), situational competency (6 items), and emotional response (4 items). Responses were recorded on a 5-point Likert Scale (1 = strongly disagree to 5 = strongly agree), with total scores ranging from 16 to 80. Higher score indicated greater satisfaction with SBL. Reverse coding was applied to necessary items before summing the scores. Content validity was evaluated by five experts, with all items receiving an I-CVI of 1.00 and the S-CVI also reaching 1.00, indicating excellent validity. Pilot testing indicated that item clarity ratings on a 4-point scale ranged from 3.9 to 4.0, suggesting that all items were generally well understood and easy to interpret. This scale has been demonstrated by Kim and Heo [20] to have good validity and reliability, with a Cronbach’s alpha value of 0.88. EFA conducted by the original developers confirmed a three-factor structure, with factor loadings ranging from 0.625 to 0.924 and a total explained variance of 66% [20]. Our EFA results also supported a three-factor structure, with factor loadings ranging from 0.435 to 0.845 and a total explained variance of 70%. In the present study, the scale exhibited strong internal consistency, with a Cronbach’s alpha of 0.87.

### Data analysis

Data analysis was performed using STATA version 16.0 (Stata Corp., College Station, TX, USA). Descriptive statistics including frequencies, proportions, means, and standard deviations (SD) were used to describe sample characteristics. To compare psychological safety, simulation design, and satisfaction with SBL between male and female students, two tailed t- were conducted with a 95% confidence level. Multivariable linear regression was employed to identify factors associated with SBL satisfaction following simulation-based assessments and to estimate the interaction effect between psychological safety and gender on satisfaction. The regression models used bias-corrected bootstrap 95% confidence intervals (CI) with 1,000 samples to improve statistical accuracy and robustness, particularly given the sample size and the potential for non-normality in residuals. Bootstrapping was chosen to provide more reliable estimates of standard errors and confidence intervals, reducing bias and improving inference stability [21]. Before conducting regression analyses, multicollinearity was assessed using variance inflation factors (VIF). A subgroup analysis was also conducted to further explore potential differences in the associations between psychological safety, simulation design features, and satisfaction across gender groups. This approach provides additional insights into potential gender-based variations in effect sizes, which may not be fully captured by an interaction model alone [22].

## Results

### Characteristics of the study sample

Table 1 presents the sample characteristics of 243 undergraduate nursing students. Among them, 36 students (14.8%) were men and 207 students (85.2%) were women. The mean age of the students was 22.5 years. The majority of the students (68.2%) were middle grade. The distribution of students across the academic years was fairly balanced: 82 students (33.7%) in 2022, 64 students (26.3%) in 2023, and 97 students (39.9%) in 2024.

**Table 1:** Sample characteristics of undergraduate nursing students

Characteristics	Nursing students (*N* = 243)
	*N* (%)
Gender	
Male	36 (14.8)
Female	207 (85.2)
Age in years^a^ (Mean, SD)	22.5 (1.8)
Grade	
High	39 (16.3)
Middle	163 (68.2)
Low	37 (15.5)
Missing	4
Survey year	
2022	82 (33.7)
2023	64 (26.3)
2024	97 (39.9)

a. Range: 21–36

### Psychological safety, simulation design features, and satisfaction with simulation-based learning by gender

The results for psychological safety, simulation design features, and satisfaction following simulation-based assessments among nursing students are shown in Table 2. Female students reported lower psychological safety (36.74 vs. 40, *p* = 0.05), particularly in interpersonal risk (7.51 vs. 8.56, *p* = 0.04) than male students. While there was no significant difference in the overall perception of simulation design features, male students reported experiencing lower fidelity in the simulations (8.05 vs. 8.57, *p* = 0.01). Satisfaction with SBL showed no significant gender differences.

**Table 2:** Gender difference in psychological safety, simulation design features, and satisfaction with current learning

	Male students (*n* = 36)	Female students (*n* = 207)	t	*p*-value
	Mean (SD)	Mean (SD)		
Psychological safety (14–70)	**40 (10.68)**	**36.74 (9.06)**	**1.93**	**0.05**
Dealing with uncertainty (4–20)	10.33 (3.82)	9.58 (3.76)	1.11	0.25
Being exposed (4–20)	11.86 (3.19)	10.95 (3.26)	1.56	0.10
Being unsupported (3–15)	9.25 (2.57)	8.71 (2.57)	1.16	0.23
Interpersonal risk (3–15)	**8.56 (3.29)**	**7.51 (2.84)**	**1.99**	**0.04**
Perceived simulation design features (21–105)	86 (9.32)	87.06 (9.42)	-0.62	0.53
Objective and information (6–30)	23.78 (2.79)	24.11 (3.21)	-0.58	0.52
Support (4–20)	15.72 (2.30)	15.92 (2.47)	-0.45	0.63
Problem solving (5–25)	20.53 (2.68)	20.58 (2.64)	-0.12	0.91
Feedback/guided reflection (4–20)	17.92 (1.83)	17.87 (2.14)	0.12	0.89
Fidelity (2–10)	**8.05 (1.51)**	**8.57 (1.14)**	**-2.39**	**0.01**
Satisfaction with simulation-based learning (16–80)	64.28 (8.32)	63.10 (8.17)	0.79	0.42
Learning content (6–30)	27.39 (3.03)	26.91 (2.82)	0.85	0.39
Situational competency (6–30)	26.44 (3.16)	26.32 (3.09)	0.21	0.83
Emotional response (4–20)	10.44 (4.16)	9.86 (4.61)	0.71	0.44

Note. Higher scores indicate greater psychological safety, better perceived simulation design features, and higher satisfaction with simulation-based learning

### Factors associated with satisfaction following simulation-based assessments

Model 1 was adjusted for gender, age, grade, survey year, psychological safety, and perceived simulation design features. SBL satisfaction following simulation-based assessments was significantly associated with both psychological safety (coefficient = 0.17, 95% CI: 0.06–0.28) and perceived simulation design features (coefficient = 0.47, 95% CI: 0.37–0.56). In Model 2, which added an interaction term between psychological safety and gender to Model 1, both psychological safety (coefficient = 0.27, 95% CI: 0.05–0.5) and perceived simulation design features (coefficient = 0.46, 95% CI: 0.36–0.57) remained significantly associated. Although the adjusted R-square value increased slightly from 0.3794 to 0.3806, no significant interaction effect between psychological safety and gender was observed, and no additional factors showed significance in Model 2 (Table 3).

**Table 3:** Factors associated with satisfaction with simulation-based learning: multivariable analysis

	Satisfaction with simulation-based learning			
	Model1^a^		Model2^b^	
	Coefficient	95% CI	Coefficient	95% CI
Female	-1.74	-4.06, 0.58	3.38	-6.63, 13.38
Age	-0.30	-0.8, 0.2	-0.31	-0.83, 0.21
Grade (ref. High)				
Middle	0.87	-1.23, 2.96	0.91	-1.19, 3.02
Low	0.32	-2.59, 3.24	0.24	-2.57, 3.05
Survey year (ref. 2024)				
2022	-0.23	-2.11, 2.97	-0.38	-2.29, 1.53
2023	0.48	-2.63, 3.31	0.48	-1.57, 2.52
Psychological safety	**0.17**	**0.06**,** 0.28**	**0.27**	**0.05**,** 0.5**
Perceived simulation design features	**0.47**	**0.37**,** 0.56**	**0.46**	**0.36**,** 0.57**
Psychological safety X Female			-0.13	-0.38, 0.12
Adjusted R-squared	0.3794		0.3806	

a. Adjusting for gender, age, grade, survey year, psychological safety, perceived simulation design features

b. Additionally adjusting for interaction between psychological safety and gender to Model1

In the subgroup analysis (Table 4), psychological safety (female: coefficient = 0.14, 95% CI: 0.03–0.25, male: coefficient = 0.35, 95% CI: 0.1–0.59) and simulation design features (female: coefficient = 0.47, 95% CI: 0.37–0.58, male: coefficient = 0.36, 95% CI: 0.08–0.65) consistently remained significant for both female and male students. No other variables showed significance in the subgroup analysis. The adjusted R-square value was higher for male students (adjR^2^ = 0.4387) than for female students (adjR^2^ = 0.3697).

**Table 4:** Factors associated with satisfaction with simulation-based learning: subgroup analysis

	Satisfaction with simulation-based learning			
	Female (*n* = 203)		Male (*n* = 36)	
	Coefficient	95% CI	Coefficient	95% CI
Age	-0.26	-0.82, 0.29	-0.17	-1.6, 1.28
Grade (ref. High)				
Middle	0.63	-1.91, 3.17	2.26	-3.49, 8.02
Low	0.52	-2.81, 3.86	-1.43	-8.28, 5.43
Psychological safety	**0.14**	**0.03**,** 0.25**	**0.35**	**0.1**,** 0.59**
Simulation design feature	**0.47**	**0.37**,** 0.58**	**0.36**	**0.08**,** 0.65**
Adjusted R-squared	0.3697		0.4387	

## Discussion

This study aimed to investigate factors linked to satisfaction, and examine the interaction of psychological safety and gender on satisfaction, including a subgroup analysis by gender following simulation-based assessment. We identified the gender differences with regards to the effects of psychological safety and simulation features on satisfaction following simulation-based assessments. Although we did not observe a significant interaction effect between gender and psychological safety on satisfaction following simulation-based assessments, a noteworthy relationship emerged between psychological safety, perceived simulation design features, and overall satisfaction.

Our findings underscore the significant association between perceived simulation design features and satisfaction following simulation-based assessments. In Jeffries’ simulation framework, simulation design is identified as one of the key factors contributing to ideal simulation experiences for nursing students [15]. The literature indicates that the achievement of expected learning outcomes is significantly influenced by simulation design features [15]. The International Nursing Association for Clinical Simulation and Learning (INACSL) has also emphasized the importance of these design features in achieving effective learning outcomes in SBL [23]. Therefore, our results suggest that students’ perception of well-established simulation design features plays an important role in their satisfaction, aligning with findings from Cho and Kim’s study on nursing students in South Korea [24]. Previous research highlights that specific simulation design features, such as clear learning objectives, perceived facilitator’s support, problem-solving opportunities, constructive feedback and level of fidelity are critical in improving student satisfaction [25–27]. Based on the research findings regarding the interaction between psychological safety, gender, and nursing students’ satisfaction with simulation-based assessments, educators can implement several actionable strategies to create a more supportive and effective learning environment.

We also observed statistically significant gender differences in the perception of simulation fidelity, despite the absence of significant differences in overall perceived simulation design scores. In the previous study, female students demonstrated better performance and attitudes with low-fidelity mannequins, while male students tended to excel with high-fidelity mannequins [28]. The physical fidelity involves the patient(s), simulator/manikin, standardized patient, equipment, embedded actors, and related props [29, 30]. The nurse educator should use psychological and, conceptual fidelity as well as physical fidelity to achieve the learner outcomes in designing the simulation based experience [31]. To effectively design a SBL experience that incorporates psychological, conceptual, and physical fidelity, the nurse educator should develop scenarios that reflect real-life situations nurses may encounter, including complex patient interactions and ethical dilemmas [31, 32]. Setting up the simulation environment to mimic a clinical setting as closely as possible, including hospital equipment, supplies, and layout could be effective to enhance the physical fidelity [32]. Enhancing realism in nursing students’ simulation education through the use of a fiction contract can be achieved. A fiction contract is an explicit agreement between educators and learners that learner engagement is dependent on both parties [33]. The educator is responsible for creating a scenario that is as real as possible within the limitations of the simulated environment [34]. Nursing educator should establish a fiction contract that outlines the roles and responsibilities of students in the pre-briefing. This helps to create a safe environment where students can immerse themselves in the scenario without fear of judgment [32].

In this study, satisfaction following simulation-based assessments increased with higher levels of psychological safety. It has been reported that there were significant association between psychological safety and satisfaction with SBL in nursing education [35]. Interestingly, female students reported feeling a lower level of psychological safety, especially when it came to interpersonal risk in this study. Women tend to be better at interpreting nonverbal communication—such as facial expressions, body language, or tone of voice—and remembering these behaviors [36]. These heightened perceptual skills may make women more aware of interpersonal risk, potentially leading to greater fear of negative feedback or poor results after simulation-based assessments compared to male students [37]. In this study, the difference may have stemmed from female students’ greater ability to pick up on interpersonal cues, which could have resulted in a stronger sense of interpersonal risk than male students in a simulation environment. Considering that women are more sensitive to interpersonal relationship, nurse educator should not only establish an informative and purposeful prebriefing session to help students understanding educational setting and the social contract they engage in, but also encourage their feeling of simulation experiences during debriefing session to create a supportive and accessible environment by building rapport with students.

The subgroup analysis in this study confirmed the consistency of psychological safety and simulation design feature across genders. This analysis was crucial to ensure that the smaller number of male students did not disproportionately influence the results or lead to exaggerated gender-specific outcomes. While psychological safety and well-designed simulation were key determinants of satisfaction for all student groups, the higher adjusted R-square for male students suggests that these two variables explained a greater proportion of the variance in satisfaction among male students compared to female students. This finding indicates that male students’ satisfaction may be more directly aligned with these predictors, while other factors could associate satisfaction levels among female students.

As the number of male nursing students continues to rise annually, understanding gender differences in psychological safety is essential for considering inclusive and supportive educational strategies. Despite the observed gender differences in psychological safety, we did not observe a significant interaction effect on satisfaction. Our subgroup analysis was deemed appropriate in this context to better reflect the underlying differences and mitigate the influence of unequal sample sizes, ensuring that the findings would be reliable. This may suggest that, despite varying levels of psychological safety, its impact is consistent across genders. Simulation-based assessments have many advantages; however, paradoxically, the stress and anxiety caused by these assessments can undermine the effectiveness of simulation training [38, 39].

This research uniquely examines how gender influences students’ perceptions of psychological safety during simulations and how this interaction impacts their overall satisfaction, providing novel insights into the role of interpersonal dynamics and gender-specific factors in SBL environments. This study result suggests the increased focus on participant-centered learning and consideration of various needs among the learners in SBL. Professional development plan should include developing the pre-briefing and debriefing skill to enhance student’s psychological safety and perceived realism in simulation design to achieve learning outcome for both male and female students. Nurse educators should recognize gender differences in learning preferences and communication styles and offer tailored support and resources that meet diverse needs, ensuring that all students feel equally valued and respected. Since women are more sensitive to interpersonal relationships, nurse educators should encourage students’ emotional expression during simulation experiences. Designing simulation scenarios that reflect diverse patient populations and experiences would not only prepare students for real-world situations but also increases engagement and satisfaction for both male and female students.

To deepen the understanding of factors associated with nursing students’ satisfaction with simulation-based assessments, particularly regarding the interaction between psychological safety and gender, future research should focus on longitudinal studies and mixed-methods approaches. By investigating these relationships over time and capturing subjective experiences, future research can inform educational strategies and policies that enhance the learning environment for nursing students. This comprehensive understanding is crucial for fostering an inclusive, supportive, and effective educational experience.

### Limitation

This study had several limitations. First, this study was conducted at a single university in South Korea, which may limit the generalizability of the findings to nursing students in other institutions or educational settings. Differences in curricula, simulation resources, and institutional culture across universities may influence students’ perceptions of psychological safety and satisfaction with SBL. Compared to lower-year students, fourth-year students have greater clinical exposure and familiarity with SBL, which may have influenced their perceptions of psychological safety, simulation design, and satisfaction. Second, although a subgroup analysis was conducted to account for potential biases, the relatively small number of male students may have limited the statistical power to detect gender-specific differences. However, this proportion closely reflects national trends, where approximately 15% of nursing students in South Korea are men [40]. Third, the study relied on self-reported measures, which are subject to reporting biases, such as social desirability and negative affectivity. Social desirability bias refers to the tendency of participants to adjust their responses to present themselves in a favorable light or to conform to perceived social norms, potentially inflating positive feedback [41]. Negative affectivity, on the other hand, reflects a predisposition toward negative emotional states, introspection, and a focus on unfavorable aspects of experiences, which may have influenced participants’ responses. These biases could have affected the validity of the findings [42]. In terms of measurements, participant characteristics were used to represent general student characteristics, which may limit generalizability. Facilitator characteristics, which may have influenced outcomes, were not measured. Additionally, the instruments used were not systematically validated, which may affect the reliability of the results. Future studies should address these issues to enhance validity and applicability. Fourth, the data used convenience sampling, which also limited data interpretation. Fifth, the cross-sectional design of the study limited our ability to determine causal relationships between the variables observed. Future research should employ a longitudinal study design to establish the causal relationship between satisfaction, psychological safety and gender following simulation-based assessments. Finally, although participation in this survey was voluntary and conducted as part of a course evaluation, students may have felt implicitly obligated to participate, which could have contributed to the 100% response rate.

## Conclusion

This study expands on prior research by providing empirical evidence of gender-specific responses to psychological safety and simulation design in SBL. The findings suggest the consideration for gender-sensitive approaches in SBL. This study might help guide educators and researchers develop effective simulation-based curriculums and provide foundational evidence for a well-designed professional development plan.

## Data Availability

The datasets used and analysed during the current study are available from the corresponding author on reasonable request.
